# Platelet release of Vascular Endothelial Growth Factor (VEGF) in patients undergoing chemotherapy for breast cancer

**DOI:** 10.1186/2040-2384-1-7

**Published:** 2009-10-24

**Authors:** Cliona C Kirwan, Gerard J Byrne, Shant Kumar, Garry McDowell

**Affiliations:** 1 Department of Academic Surgery, South Manchester University Hospitals NHS Trust, Wythenshawe Hospital, Southmoor Road, Manchester, M23 9LT, UK; 2 Department of Pathology, University of Manchester and Christie Hospital, Manchester Medical School, Oxford Road, Manchester, M13 9PT, UK; 3 British Institute of Technology and E-Commerce, Romford Road, London, E7 9HZ, UK

## Abstract

**Background:**

Venous thromboembolism (VTE) following breast cancer chemotherapy is common. Chemotherapy-induced alterations in markers of haemostasis occur during chemotherapy. In this study we investigated the changes in serum and plasma VEGF, together with platelet release of VEGF and related these to the development of VTE at 3 months.

**Methods:**

Serum and plasma VEGF, together with platelet release of VEGF were measured prior to chemotherapy and at 24 hours; four-, eight days and three months following commencement of chemotherapy in early and advanced breast cancer patients and in age and sex matched controls. Duplex ultrasound imaging was performed after one month or if symptomatic.

**Results:**

Of 123 patients 9.8% developed VTE within three months. Serum and plasma VEGF were increased in advanced breast cancer as was platelet release of VEGF. Prior to chemotherapy a 100 μg/ml increase in serum VEGF was associated with a 40% increased risk of VTE, while a 10 μg/ml increase in plasma VEGF was associated with a 20% increased risk of VTE. Serum VEGF showed a different response to chemotherapy in those who developed VTE.

**Conclusion:**

A group of patients at risk of VTE could be identified, allowing targeted thrombopropylaxis. Whether or not the response in VEGF during chemotherapy has any angiogenic significance remains to be elucidated.

## Introduction

Venous thromboembolism (VTE) following breast cancer chemotherapy is not uncommon. In early breast cancer, VTE occurs in 5-10% of patients receiving chemotherapy [1-3], with a mortality of 0.2-0.5% [1,4]. VTE rises to approximately 18% in advanced breast cancer [5], with a mortality of 9% [5]. Approximately two thirds of all venous thromboemboli occur within three months of commencing chemotherapy [3,6], however despite this, thromboprophylaxis is rarely used [7].

Previous work has demonstrated a hypercoagulable state in breast cancer patients, with elevated markers of coagulation, including thrombin-antithrombin (TAT) [8,9], fibrinogen [10], D-dimer [11,12] and tissue factor (TF) [13,14].

Several small studies have reported alterations in markers of coagulation in response to breast cancer chemotherapy, which support the development of a chemotherapy-induced hypercoagulable state [15-19]. Several pathogenic mechanisms have been suggested such as increased expression or release of procoagulants and cytokines from damaged cells, a direct toxic effect on vascular endothelium or upregulation of platelet or monocyte activity.

The requirement for platelets in cancer metastasis was recognised about 30 years ago and it is now recognised that platelets are an integral part of the microthrombus that is thought to promote the arrest and lodgement of circulating tumour cells [20-22].

Platelets are numerous in the circulation and contain large stores of factors known to be essential for angiogenesis. Vascular endothelial growth factor (VEGF) is one such protein that is stored in large quantities in platelet α-granules [23] and elevated circulating levels have been observed in patients with malignancy including breast cancer [24-26]. VEGF is pro-angiogenic and it has been shown that VEGF secreted by megakaryocytes may contribute to proliferation of vascular endothelial cells [27]. Our previous study has shown that platelets from breast cancer patients release more VEGF per platelet than normal controls [28]

We hypothesized that platelets are functionally altered in women with breast cancer and this may represent a possible pathophysiological mechanism for the observed VTE during chemotherapy. In the current study we prospectively followed early and advanced breast cancer patients commencing chemotherapy to establish early alterations in the circulating plasma and serum levels of VEGF together with platelet release of VEGF.

## Materials and methods

### Patients

A total of 123 female patients [median age 52 (range, 31-78) years] commencing chemotherapy for breast cancer were recruited. Of these, 87 were receiving adjuvant chemotherapy following curative surgery, and 36 receiving chemotherapy for radiographically-proven metastatic breast disease (Table 1).

**Table 1:** Chemotherapy regimens used in breast cancer patients

Chemotherapy Regimen	Number of patients
*Adjuvant Regimens*	
5-fluorouracil, epirubicin, cyclophosphamide	65
Cyclophosphamide, methotrexate, 5-fluorouracil	15

Epirubicin, cyclophosphamide	4

Epirubicin	3

*Metastatic Regimens*	

Docetaxol	15

Cyclophosphamide, methotrexate, 5-fluorouracil	8

Epirubicin, docetaxol	6

Vinorelbine, mitomycin	3
Epirubicin	2
5-fluorouracil, epirubicin, cyclophosphamide	1
Vinorelbine, 5-fluorouracil	1

### Control subjects

Sixty eight age matched female controls [median age 48 (range, 31-78) years], with no history of cancer acted as control subjects.

### Protocol

A prospective cohort study was undertaken. Serum vascular endothelial growth factor [sVEGF] and plasma vascular endothelial growth factor [pVEGF]) were measured prior to chemotherapy and at 24 hours, four-, eight days and three months following commencement of chemotherapy in all patients. A clinical assessment for VTE was performed at the same time points. Duplex ultrasound imaging was performed one month following commencement of chemotherapy or if symptoms developed.

### Blood sampling and analytical methods

Atraumatic venous blood sampling was performed at the antecubital fossa and all specimens separated and stored within two hours after being collected into tubes containing citrate as anticoagulant. Citrate samples were immediately taken onto ice whilst serum samples were allowed to clot at room temperature. All samples were centrifuged at 2500 g for 20 minutes at 4°C and the plasma or serum decanted before being stored at -80°C in 300 μl aliquots. Platelet depleted plasma was prepared for the analysis of VEGF as detailed: Citrated tubes were immediately plunged into ice and taken to the laboratory were the sample was centrifuged at 4°C for twenty minutes at 3500 g. The supernatant was removed and re-centrifuged for 20 mins at 3500 g at 4°C following which the platelet depleted plasma (PDP) was aliquoted and the last 0.5 ml discarded. All samples were stored at -80°C until analysis.

Platelet count was measured using the Advia 120 Haematology System (Bayer Diagnostic, UK)

### VEGF Assay

Serum and platelet depleted plasma VEGF were analysed using an enzyme-linked immunosorbent assay (ELISA) by R&D Systems^®^, Oxon, UK, with a sensitivity of 9 μg/ml.

In duplicate, sample (100 μl) diluted with 100 μl of assay diluent was incubated with the capture antibody for 2 hours at room temperature. Following washing steps (×3), 200 μl of detection antibody conjugate was added and the assay incubated for 2 hours at room temperature. The plate was again washed (×3) and the detection substrate added and incubated for 25 minutes at room temperature. Stop solution (25 μl) was added and the optical density read at 450 nm.

### Platelet VEGF

The amount of VEGF released per platelet was calculated using the following previously validated formula [29]

### Ethical approval

The study was approved by the South Manchester Local Research Ethics Committee and all patients gave written informed consent.

### Statistical methods

Data on sVEGF and pVEGF was parametric after log conversion and so reported as geometric mean (confidence interval). Platelet releasate VEGF concentration was expressed as median (range). Comparative group analysis (early, advanced breast cancer and controls) of pre-chemotherapy patient values was by ANOVA, with further analysis of subgroups using Scheffe. Comparative group analysis (VTE within three months, VTE free) of patient values was by independent T-test. Changes in patient serum or plasma values with chemotherapy as compared with pre-treatment values were performed by paired T test, however to minimise errors induced by multiple tests a repeated measures analysis (Greenhouse Geiser correction) to compare trends over time in patients with and without VTE was used. Comparative group analysis (VTE within three months, VTE free) of changes in coagulation parameters with chemotherapy as compared with pre-treatment values were performed by analysis of covariance. A significance of p < 0.05 was used. Binary logistic regression to identify predictors of VTE was also performed. Analysis was performed on baseline data, and change from baseline. Appropriate corrections were made for cancer stage and age.

## Results

Of 123 breast cancer patients receiving chemotherapy, 12 (9.8%) developed VTE within 3 months of chemotherapy, of which eight (66.7%) were symptomatic. Six of 36 (17%) metastatic breast cancer patients and six of 87 (6.9%) early breast cancer paients receiving adjuvant chemotherapy developed VTE.

### Baseline data: prior to chemotherapy

Prior to chemotherapy, serum and plasma VEGF were all increased in advanced breast cancer patients compared to controls. Further serum and plasma VEGF were increased in advanced breast cancer compared to early breast cancer. Platelet count was increased in advanced breast cancer compared to controls. Calculated VEGF release per platelet from advanced cancer patients was elevated compared to early breast cancer patients and the control group (Table 2).

**Table 2:** Baseline biomarker results prior to chemotherapy

*Coagulation marker*	*Advanced breast cancer*	*Early breast cancer*	*Control*	*p ANOVA (Scheffe-showing paired comparisons)*
sVEGF μg/ml, geometric mean (CI)***[n]***	344.0 (270.7-437.2)*† **[35]**	181.0 (155.9-210.2)* ***[86]***	28.8 (25.3-32.8)*† **[61]***	*<0.001* (*<0.001, †<0.001)

pVEGF μg/ml, geometric mean (CI)***[n]***	22.8 (17.6-29.4)*† **[35]**	14.2 (12.1-16.6)* **[86]**	15.1 (12.7-17.9)*† **[61]***	*0.004* (*0.01, †0.03)

Platelet count × 10^9^/l, mean (CI) ***[n]***	326.7* (286.9-366.5) ***[36]***	309.6 (293.0-326.2) ***[87]***	278.6 (257.7-299.6)* ***[45]***	0.04 *0.05

Platelet VEGF VEGF μg/ml per platelet × 10^9^ median (range) ***[n]***	1.02*†‡ (0.17-3.53) ***[35]***	0.53* (0.05-2.89) ***[83]***	0.53 ‡(0.007-2.48) ***[45]***	*0.05* (*<0.001, †0.008, *‡0.002*

Analysis of the difference between groups used Analysis of Variance (ANOVA). Where differences were found, further analysis (between pairs of groups, with respective pairs for each molecule marked with *†*‡*§ was performed using Scheffe. (95% Confidence interval (CI)). sVEGF = serum VEGF; pVEGF = plasma VEGF

### Baseline data: Prior to chemotherapy - Development of VTE

Prior to chemotherapy plasma VEGF was significantly higher in those who developed VTE within 3 months of commencing chemotherapy compared to those who did not (27.8 [14.3-54.1] vs 15.4 [13.5-17.7] μg/ml, p = 0.01 [independent T-test]). Interestingly serum VEGF was not significantly different between both groups (282 [136.5-585.3] vs 212 [185.6-243.1] μg/ml, p = 0.2 [independent T-test]) In addition, platelet release of VEGF did not demonstrate a significant difference in those who developed VTE within 3 months compared to those who did not. (0.9 [0.4-1.9] vs 0.6 [0.5-0.7] VEGF μg/ml per platelet × 10^9^, median [range], p = 0.6 [independent T-test]). Prior to chemotherapy however a 100 μg/ml increase in sVEGF was associated with a 40% increased risk of VTE (P = 0.003). A 10 μg/ml increase in pVEGF was associated with a 20% increased risk of VTE (P = 0.007)

### Response to chemotherapy

The mean or geometric mean (CI) of plasma and serum VEGF together with platelet releasate VEGF (median (range)) at baseline, 24 hours, four and eight days and three months following chemotherapy are given in table 3, and for patients sub-divided into those developing VTE and remaining free of VTE in tables 4 and 5 respectively.

**Table 3:** Alterations in biomarker parameters induced by chemotherapy in breast cancer patients

*Procoagulant/adhesion molecule*	*Pre-chemo [n]*	*Day 1 (post) [n]*	*Day 4 (post) [n]*	*Day 8 (post) [n]*	*3 months (post) [n]*	*Trend over time (repeated meas, GG)*
sVEGF μg/ml, geometric mean (CI)	218.0 (190.3-249.6) ***[121]***	203.4 (176.5-234.4) ***[114]***	158.3 (136.6-183.4) ***[114]***	154.3 (132.5-179.8) ***[116]***	236.7 (205.0-273.4) ***[109]***	*p < 0.001*

pVEGF μg/ml, geometric mean (CI)	16.4 (14.2-18.8) ***[120]***	14.9 (13.2-16.9) ***[115]***	16.3 (14.0-18.0) ***[112]***	20.5 (18.3-13.0) ***[114]***	21.6 (18.8-24.9) ***[106]***	*p < 0.001*

Platelet count × 10^9^/l Mean (CI)	314 (298.4-330.9) **[123]**	306 (287.2-325) **[99]**	263.3 (249.7-276.8) **[108]**	242.3 (227.3-257.2) **[117]**	310 (285.0-334) **[112]**	*P < 0.001*

Platelet VEGF VEGF μg/ml per platelet × 10^9^ median (range) **[n]**	0.62 (0.54-0.72) **[120]**	0.61 (0.52-0.71) **[99]**	0.51 (0.43-0.61) **[108]**	0.53 (0.45-0.64) **[114**]	0.72 (0.62-0.83) **[106]**	*P < 0.001*

**Table 4:** Alterations in biomarker parameters induced by chemotherapy in breast cancer patients developing VTE within three months of chemotherapy

*Coagulation marker*	*Pre-chemo [n]*	*Day 1 (post chemo) [n]*	*Day 4 (post chemo) [n]*	*Day 8 (post chemo) [n]*	*3 months (post chemo) [n]*
sVEGF μg/ml, geometric mean (CI)	282.6 (136.5-585.3) ***[11]***	267.8 (136.5-525.4) ***[11]***	220.1 (110.7-437.3) ***[11]***	226.0 (116.3-439.2) ***[11]***	358.8 (116.3-774.2) ***[9]***

pVEGF μg/ml, geometric mean (CI)	27.8 (14.3-54.1) ***[12]***	19.5 (10.4-36.6) ***[11]***	19.0 (9.4-38.3) ***[11]***	23.4 (13.2-41.5) ***[11]***	18.7 (13.7-25.5) ***[9]***

Platelet count × 10^9^/l mean (CI)	342.5 (265.8-419.2) **[12]**	338.9 (246.4-431.4) **[10]**	269.6 (225.5-313.8) **[11]**	274 (216.8-331.2) **[11]**	386.2 (238.3-534.1) **[10]**

Platelet VEGF VEGF μg/ml per platelet × 10^9^ median (range) **[n]**	0.89 (0.42-1.87) **[11]**	0.89 (0.47-1.68) **[10]**	0.86 (0.39-1.86) **[11]**	0.83 (0.39-1.75) **[11]**	1.02 (0.53-1.97) **[9]**

**Table 5:** Alterations in biomarkers induced by chemotherapy in breast cancer patients remaining free of VTE within three months of chemotherapy

*Coagulation marker*	*Pre-chemo [n]*	*Day 1 (post chemo) [n]*	*Day 4 (post chemo) [n]*	*Day 8 (post chemo) [n]*	*3 months (post chemo) [n]*
sVEGF μg/ml, geometric mean (CI)	212.4 (185.6-243.1) ***[110]***	197.5 (171.1-228.0) ***[103]***	152.8 (131.7-177.4) ***[103]***	148.3 (126.8-173.3) ***[105]***	228.0 (197.4-263.4) ***[100]***

pVEGF μg/ml, geometric mean (CI)	15.4 (13.5-17.7) ***[108]***	14.5 (12.9-16.4) ***[104]***	16.0 (13.7-18.7) ***[101]***	20.2 (18.0-22.7) ***[103]***	21.9 (18.8-25.5) ***[97]***

Platelet count × 10^9^/l, mean (CI)	311.6 (295.2-328) **[111]**	302.4 (283.4-321.4) **[89]**	262.5 (248.1-277) **[97]**	239 (223.4-254.5) **[106]**	302.5 (279.6-325.5) **[102]**

Platelet VEGF VEGF μg/ml per platelet × 10^9^ median (range) **[n]**	0.61 (0.52-0.71) **[108]**	0.58 (049-0.69) **[89]**	0.49 (0.41-0.58) **[97]**	0.52 (0.43-0.62) **[103]**	0.70 (0.61-0.81) **[97]**

Analysing all patients together, irrespective of subsequent development of VTE, serum and plasma VEGF, together with platelet release of VEGF showed a significant trend over time (repeated measures analysis). Serum VEGF decreased during the first 8 days, however plasma VEGF increased (P < 0.001). By 3 months both showed trend for increased levels (P < 0.001). In patients with and without VTE, by four days following chemotherapy, platelet count was reduced, however it remained within normal limits. Levels returned to baseline by three months (Table 3).

A differing pattern of change was observed in serum VEGF between advanced and early breast cancer patients which was not reflected in plasma VEGF. The difference was apparent in the first eight days following chemotherapy, where sVEGF levels in advanced cancer showed a greater decrease, from baseline, at 24 hours (p = 0.004) and four days (p = 0.002) compared to early breast cancer. The difference between the trends seen in response to chemotherapy in advanced and early breast cancer patients was no longer apparent by three months (p = 0.2). The rapid decrease in sVEGF levels, in advanced breast cancer, as an early response to chemotherapy was not mirrored by pVEGF levels. (Table 6).

**Table 6:** Table showing the chemotherapy induced changes in serum VEGF and platelet release of VEGF in advanced and early breast cancer

Procoagulant	Group	Baseline	Day 1	Day 4	Day 8	3 Months	Significant Difference in Trend i) pre-chemo to day 8 ii)Pre-chemo to 3 months
**sVEGF** μg/ml Geometric Mean (CI) **[n]**	ABC	344 (271-437) **[35]**	287 (219-376) **[33]**	240 (182-317) **[33]**	253 (196-327) **[33]**	344 (248-477) **[26]**	i) 0.01* ii) 0.2*
	
	EBC	181 (156-210) **[86]**	177 (151-207) **[81]**	134 (114-157) **[81]**	127 (107-151) **[83]**	211 (181-246) **[83]**	i) 0.01** ii) 0.2**

**VEGF released** **per platelet** μg/ml/platelet Geometric mean (CI) **[n]**	ABC	1.02 (0.8-1.30) **[35]**	0.92 (0.68-1.23) **[28]**	0.85 (0.61-1.17) **[30]**	0.92 (0.72-1.19) **[31]**	1.03 (0.77-1.39) **[25]**	i) 0.007* II) 0.03*
	
	EBC	0.53 (0.45-0.62) **[82**]	0.51 (0.42-0.63) **[67]**	0.43 (0.35-0.52) **[73**]	0.43 (0.34-0. 53) **[78]**	0.65 (0.54-0.77) **[78]**	i) 0.007** ii) 0.03**

sVEGF = serum vascular endothelial growth factor (VEGF)

ABC = advanced breast cancer

EBC = early breast cancer

*Significant difference in trend compared to early breast cancer

**Significant difference in trend compared to advanced breast cancer

Platelet release of VEGF showed a significant trend in reponse to chemotherapy. There was a decline in platelet release of VEGF by day 4 and 8 (P < 0.001) increasing to above pre-chemotherapy levels by 3 months (Table 3).

The alteration in platelet release of VEGF was significantly different in advanced breast cancer (Table 6). Although both groups appeared to show a decline in platelet release of VEGF initially following chemotherapy this was not significant in advanced cancer even at 24 hours (P = 0.15), compared to early cancer (P < 0.001 at day 8)

When patients who subsequently developed VTE were compared to patients who remained free of VTE (Tables 4 and 5), serum VEGF showed marked alterations in response to chemotherapy in those with and without VTE at three months. Serum VEGF shows a more marked increase in patients subsequently developing VTE (p = 0.01), even when corrections are made for cancer stage (Tables 4 and 5). There was a marked decrease in pVEGF, but not sVEGF, within 24 hours of commencing chemotherapy, in patients developing VTE compared to patients remaining free of VTE (change from pre-chemotherapy levels to 24 hours: -8.3 and -0.9 μg/ml, n = 11 and 104, p = 0.04). A decrease in pVEGF within 24 hours was associated with an increased risk of VTE, with a 10 μg/ml decrease from baseline, at 24 hours, increasing the risk of VTE by 25% (p = 0.05).

Platelet release of VEGF during chemotherapy did not demonstrate a significantly different response in those who developed VTE within 3 months compared to those who did not (P = 0.6).

## Discussion

Consistent with previously published literature, sVEGF and pVEGF levels in this study are significantly elevated in advanced breast cancer patients [29]. Interestingly, pVEGF levels in the early breast cancer group (following apparent complete surgical resection) are comparable to controls. Previous literature report increased levels in early breast cancer patients prior to surgery [30,31].

The higher VEGF seen in serum compared to plasma samples, is consistent with previous studies reporting platelet release of VEGF as the main source of serum VEGF [32,33]. The much lower levels of pVEGF may represent ongoing platelet release of VEGF in addition to other sources, including tumour cells. Consistent with previously published literature, sVEGF and pVEGF levels in this study are significantly elevated in advanced breast cancer patients [29]. Several studies report elevated serum and plasma VEGF in early cancer patients, prior to curative surgery [29,34].

In the first eight days following chemotherapy, the increase in pVEGF is matched by a decrease in sVEGF. This may reflect increased platelet release of VEGF as a response to chemotherapy, with a consequent reduction in stored levels of VEGF in the platelet α-granules [35]. This is supported by the reduction in calculated VEGF per platelet in the eight days following chemotherapy. The decrease seen in serum VEGF is particularly marked in advanced cancer (both absolute sVEGF, and VEGF released per platelet). This implies the platelet response to chemotherapy is altered in the presence of cancer. As platelet content of VEGF increases, this must be either due to increased platelet (megakaryocyte) production of VEGF [36] or platelet scavenging of circulating VEGF produced from other cellular sources [37,38]. The lack of difference between advanced and early breast cancer response to chemotherapy may provide evidence to refute previous theories that increased platelet content of VEGF in cancer is due to platelet scavenging of tumour VEGF [35,39].

Previous research has shown that platelet lysates from breast cancer patients have increased VEGF concentrations compared to controls [39]. In this study we show that this is limited to advanced cancer patients. A previous study by Salgado and colleagues shows increased VEGF release per platelet in advanced cancer compared to early cancer (prior to surgery), and early cancer compared to control. However in their study they fail to correct for background circulating VEGF (pVEGF) [35]. Previous work from our group [28] showed VEGF release from platelets was also greater in early breast cancer patients compared to controls, but did not study advanced breast cancer patients, although this study used a different methodology and smaller number of patients.

After an initial decrease in platelet VEGF content, possibly due to VEGF release stimulated by chemotherapy, the possible increase in platelet VEGF content in advanced cancer only, suggests an important store of this angiogenic molecule in active cancer. The profound effect of chemotherapy on platelet VEGF content in early breast cancer suggests an initial early release of VEGF by platelets in response to chemotherapy, hence depleting stores. This appears to be followed by a reactive increase in VEGF storage, which is mirrored by the absolute platelet count. Verheul and co-workers, in a study of 27 breast cancer patients receiving chemotherapy, reported an initial thrombocytopenia followed by a strong platelet rebound coinciding closely with a sVEGF peak. However, by not correcting this for background (plasma) levels of VEGF, or calculating VEGF per platelet, they concluded that sVEGF simply reflects platelet count [40]. The data from this present study suggests not just a chemotherapy-induced rebound in platelet count (ie decrease, followed by steady increase), but also a similar decrease and then steady increase in individual platelet content of VEGF, irrespective of platelet count. This implies an alteration in platelet function. The significance of this in relation to angiogenesis however remains unknown.

Prior to chemotherapy, plasma VEGF was increased in patients subsequently developing VTE, with a trend for increased sVEGF levels in the VTE patient group. Other investigators have reported sVEGF and pVEGF correlate with the haemostatic markers TAT, fibrinogen and D-dimer [41]. Kim and co-workers report an association between increased VEGF per platelet and portal vein thrombosis in hepatocellular carcinoma, however in this study the authors do not correct for background (plasma) VEGF levels that may contribute to the reported sVEGF per platelet levels [42]. As previously discussed, sVEGF largely consists of VEGF released from platelets, but pVEGF may also represent a significant tumour contribution. The strong association of pVEGF but not sVEGF with VTE may reflect patients with a greater tumour release of VEGF having an increased risk of VTE.

Of the procoagulants we have studied, only pVEGF demonstrates an early altered response to chemotherapy in patients developing VTE, compared to those without VTE. The marked reduction in pVEGF, but not sVEGF at 24 hours in the VTE group, suggests rapid uptake of background VEGF in the hypercoagulable patients. Whether this uptake is by cancer cells, or perhaps more likely, by altered function of platelets, remains to be established.

There was no difference in pre-chemotherapy platelet count, or platelet release of VEGF, in all breast cancer patients with and without subsequent VTE. This supports the findings of Leibovitch, who although reporting reactive thrombocytosis as a common finding following major urological surgery, found no association with thromboembolic complications [43]. Pedersen also found no association between thrombocytosis in primary lung cancer, and development of VTE [44]. However, thrombocytosis, above the normal range, occurred in the advanced breast cancer patients that subsequently developed VTE. Goodnough, in 159 advanced breast cancer patients, found no incidence of thrombocytosis, prior to chemotherapy, in patients developing VTE compared to patients without VTE. However in this analysis, Goodnough *et al *[5] included all patients developing VTE, even patients with VTE occurring after completion of treatment. Elevated levels of platelet microparticles (which correlate with platelet activation) *in vivo *have been reported for patients with activated coagulation and fibrinolysis [45], however research is minimal on platelet release of angiogenic molecules as a predictor of VTE.

In conclusion, raised pVEGF, prior to chemotherapy in patients developing VTE, may point to the procoagulant trigger for cancer-induced VTE. The increased baseline pVEGF, but not sVEGF, in patients developing VTE implies tumour VEGF rather than platelet VEGF is the important procoagulant here. However, the rapid reduction in pVEGF following chemotherapy, in the VTE group, suggests an increased VEGF uptake, either by tumour, or perhaps more likely, by platelets. This study provides evidence that a clinically useful profile could be produced to identify patients at increased risk of VTE. With such a profile, a trial of targeted anticoagulant prophylaxis could be developed.

## Competing interests

The authors declare that they have no competing interests.

## Authors' contributions

CCK: Designed the study, undertook patient recruitment, laboratory analysis and data analysis. Also prepared and critically reviewed the draft manuscript. Read and approved the final manuscript.

GJB: Designed the study, reviewed the data and critically reviewed the manuscript. Read and approved the final manuscript.

SK: Critically reviewed the manuscript and made important changes to the scientific content. Read and approved the final manuscript.

GMcD: Supervised the laboratory analysis and analysis of the data. Drafted and critically reviewed the manuscript for scientific content. Read and approved the final manuscript.

## References

[B1] Weiss RB Tormey DC Holland JF Weinberg VE Venous thrombosis during multimodal treatment of primary breast carcinoma Cancer Treat Rep 1981 65 677 679 7248984

[B2] Levine MN Gent M Hirsh J Armold A Goodyear MD Hryniuk W DePauw S The thrombogenic effect of anticancer drug therapy in women with stage II breast cancer N Engl J Med 1988 318 404 407 3340118 10.1056/NEJM198802183180703

[B3] von Tempelhoff GF Dietrich M Hommel G Heilmann L Blood coagulation during adjuvant epirubicin/cyclophosphamide chemotherapy in patients with primary operable breast cancer J Clin Oncol 1996 14 2560 2568 8823336 10.1200/JCO.1996.14.9.2560

[B4] Clahsen PC Velde CJ van de Julien JP Floiras JL Mignolet FY Thromboembolic complications after perioperative chemotherapy in women with early breast cancer: a European Organization for Research and Treatment of Cancer Breast Cancer Cooperative Group study J Clin Oncol 1994 12 1266 1271 8201388 10.1200/JCO.1994.12.6.1266

[B5] Goodnough LT Saito H Manni A Jones PK Pearson OH Increased incidence of thromboembolism in stage IV breast cancer patients treated with a five-drug chemotherapy regimen. A study of 159 patients Cancer 1984 54 1264 1268 10.1002/1097-0142(19841001)54:7<1264::AID-CNCR2820540706>3.0.CO;2-R 6547874

[B6] Seward J Byrne GJ Howell A Bundred NJ McCollum CN Does cytotoxic chemotherapy precipitate venous thromboembolism in patients with cancer Breast Cancer Res Treat 1999 57 57

[B7] Kirwan CC Nath E Byrne GJ McCollum CN Prophylaxis for venous thromboembolism during treatment for cancer: questionnaire survey Br Med J 2003 327 597 598 10.1136/bmj.327.7415.597 PMC194089 12969928

[B8] Falanga A Lwvine MN Consonni E Gritti G Delaini F Oldani E Julian JA Barbui T The effect of very-low-dose warfarin on markers of hypercoagulation in metastatic breast cancer: results from a randomized trial Thromb Haemost 1998 79 23 27 9459316

[B9] Ozyilkan O Baltali E Ozdemir O Tekuzman G Kirazli S Firat D Haemostatic changes; plasma levels of alpha2-antiplasmin-plasmin complex and thrombin-antithrombin III complex in female breast cancer Tumori 1998 84 364 367 9678618 10.1177/030089169808400310

[B10] Miller B Heilmann L Hemorheologic variables in breast cancer patients at the time of diagnosis and during treatment Cancer 1988 62 350 354 10.1002/1097-0142(19880715)62:2<350::AID-CNCR2820620220>3.0.CO;2-W 3383135

[B11] Blackwell K Haroon Z Broadwater G Berry D Harris L Iglehart JD Dewhirst M Greenberg C Plasma D-dimer levels in operable breast cancer patients correlate with clinical stage and axillary lymph node status J Clin Oncol 2000 18 600 608 10653875 10.1200/JCO.2000.18.3.600

[B12] Oberhoff C Rollwagen C Tauchert AM Hoffman O Winkler UH Schindler AE Perioperative development of a thrombogenic risk profile in patients with carcinomas of the breast: a cause of increased thrombosis Eur J Gynaecol Oncol 2000 21 560 568 11214610

[B13] Lwaleed BA Chisholm M Francis JL Urinary tissue factor levels in patients with breast and colorectal cancer J Pathol 1999 187 291 294 10.1002/(SICI)1096-9896(199902)187:3<291::AID-PATH213>3.0.CO;2-8 10398081

[B14] Ueno T Toi M Koike M Nakamura S Tominaga T Tissue factor expression in breast cancer tissues: its correlation with prognosis and plasma concentration Br J Cancer 2000 83 164 170 10.1054/bjoc.2000.1272 10901365 PMC2363475

[B15] Feffer SE Carmosino LS Fox RL Acquired protein C deficiency in patients with breast cancer receiving cyclophosphamide, methotrexate, and 5-fluorouracil Cancer 1989 63 1303 1307 10.1002/1097-0142(19890401)63:7<1303::AID-CNCR2820630713>3.0.CO;2-F 2920359

[B16] Rogers JS Murgo AJ Fontana JA Raich PC Chemotherapy for breast cancer decreases plasma protein C and protein S J Clin Oncol 1988 6 276 281 2963094 10.1200/JCO.1988.6.2.276

[B17] Canobbio L Fassio T Ardizzon A Bruzzi P Queirolo MA Zarcone D DiGeorge F Rosso R Santi L Hypercoagulable state induced by cytostatic drugs in stage II breast cancer patients Cancer 1986 58 1032 1036 10.1002/1097-0142(19860901)58:5<1032::AID-CNCR2820580509>3.0.CO;2-U 3731037

[B18] Rella C Coviello M Giotta F Maiello E Colavito P Colangelo D Quaranta M Colucci G Schittuli F A prothrombotic state in breast cancer patients treated with adjuvant chemotherapy Breast Cancer Res Treat 1996 40 151 159 10.1007/BF01806210 8879681

[B19] Pectasides D Tsavdaridis D Aggouridaki C Tsavdaridis V Visvikis A Tsatalas K Fountzilas G Effects on blood coagulation of adjuvant CNF (cyclophosphamide, novantrone, 5-fluorouracil) chemotherapy in stage II breast cancer patients Anticancer Res 1999 19 3521 3526 10629646

[B20] Trikha M Nakada MT Platelets and cancer: Implications for antiangiogenic therapy Semin Thrombo Haemostas 2002 28 39 44 10.1055/s-2002-20563 11885024

[B21] Hejna M Raderer M Zielinski CC Inhibition of metastases by anticoagulants J Natl Cancer Inst 1999 91 22 36 10.1093/jnci/91.1.22 9890167

[B22] Tang DG Honn KV Adhesion molecules and tumour metastasis: an update. Invasion Metastasis 1994 14 109 122 7657506

[B23] Ferrara N Davis-Smyth T The biology of vascular endothelial growth factor Endocrine Rev 1997 18 4 21 10.1210/er.18.1.4 9034784

[B24] Ferrari G Scagliotti GV Serum vascular endothelial growth factor levels in non-small cell lung carcinoma Eur J Cancer 1996 32A 2369 2370 9038626 10.1016/s0959-8049(96)00272-9

[B25] Dirix LY Vermeulen PB Pawinski A Prove A Benoy I DePooter C Martin M Van Oosteron AT Elevated levels of angiogenic cytokines basic fibroblast growth factor and vascular endothelial growth factor in sera of cancer patients Br J Cancer 1997 76 238 243 9231925 10.1038/bjc.1997.368 PMC2223937

[B26] Oehler MK Carrier H Diagnostic value of serum VEGF in women with ovarian tumours Anticancer Res 1999 19 2519 2522 10470186

[B27] Mohle R Green D Moore MAS Nachman RL Rafii S Constitutive production and thrombin induced release of vascular endothelial growth factor by human megakaryocytes and platelets Proc Natl Acad Sci 1997 94 663 668 10.1073/pnas.94.2.663 9012841 PMC19570

[B28] McDowell G Temple I Li C Kirwan CC Bundred NJ McCollum CN Burton IE Kumar S Byrne GJ Alteration in platelet function in patients with early breast cancer Anticancer Res 2005 25 3963 3966 16309184

[B29] Adams JA Carder PJ Downey S Forbes MA MacLennan K Allgar V Kaufmann S Hallam S Bicknell R Walker JJ Cairnduff F Selby PJ Perren TJ Lansdown M Banks RE Vascular endothelial growth factor in breast cancer: comparison of plasma, serum and tissue VEGF and microvessel density and effects of tamoxifen Cancer Res 2000 60 2898 2905 10850435

[B30] Heer K Kumar H Read JR Fox JN Monson JR Kerin MJ Serum vascular endothelial growth factor in breast cancer: its relation with cancer type and estrogen receptor status Clin Cancer Res 2001 7 3491 3494 11705867

[B31] Byrne GJ McDowell G Agarawal R Sinha G Kumar S Bundred NJ Serum vascular endothelial growth factor in breast cancer Anticancer Res 2007 27 3481 3487 17972505

[B32] Banks RE Forbes MA Kinsey SE Stanley A Ingham E Walters C Selby PJ Release of angiogenic cytokine vascular endothelial growth factor (VEGF) from platelets: significance for VEGF measurement and cancer biology Br J Cancer 1998 77 956 964 9528841 10.1038/bjc.1998.158 PMC2150108

[B33] Salgado R Vermeulen PB Benoy I Weytjens R Huget P Van Marck E Dirix LY Platelet number and interleukin-6 correlate with VEGF but not with bFGF serum levels of advanced cancer patients Br J Cancer 1999 80 892 897 10.1038/sj.bjc.6690437 10360671 PMC2362300

[B34] Belgore FM Lip GY Bareford D Wadley M Stonelake P Blann AD Plasma levels of vascular endothelial growth factor (VEGF) and its receptor, Flt-1, in haematological cancers: a comparison with breast cancer Am J Haematol 2001 66 59 61 10.1002/1096-8652(200101)66:1<59::AID-AJH1011>3.0.CO;2-Z 11426496

[B35] Salgado R Benoy I Bogers J Weytjens R Vermeulen P Dirix L Van Marck E Platelets and vascular endothelial growth factor (VEGF): a morphological and functional study Angiogenesis 2001 4 37 43 10.1023/A:1016611230747 11824377

[B36] Mohle R Green D Moore MA Nachman RL Rafii S Constitutive production and thrombin induced release of vascular endothelial growth factor by human megakaryocytes and platelets Proc Natl Acad Sci 1997 94 663 668 10.1073/pnas.94.2.663 9012841 PMC19570

[B37] Vermeulen P Salven P Benoy I Gasparini G Dirix LY Blood platelets and serum VEGF in cancer patients Br J Cancer 1999 79 370 373 10.1038/sj.bjc.6690051 9888483 PMC2362208

[B38] Caine GJ Lip GY Blann AD Platelet derived VEGF, Flt-1, angiopoietin-1 and P-selectin in breast and prostate cancer: further evidence for a role of platelets in tumour angiogenesis Ann Med 2004 36 273 277 10.1080/07853890410026098 15224653

[B39] George ML Eccles SA Tutton MG Abulafi AM Swift RI Correlation of plasma and serum vascular endothelial growth factor levels with platelet count in colorectal cancer: clinical evidence of platelet scavenging Clin Cancer Res 2000 6 3147 3152 10955796

[B40] Verheul HM Hoekman K Luykx-Bakker S Platelet transporter of vascular endothelial growth factor Clin Cancer Res 1997 3 2187 2190 9815613

[B41] Matsuyama W Hashiguchi T Mizoguchi A Iwami F Kawabata M Arimura K Osame M Serum levels of vascular endothelial growth factor dependent on the stage progression of lung cancer Chest 2000 118 948 951 10.1378/chest.118.4.948 11035661

[B42] Kim SJ Choi IK Park KH Yoon SY Oh SC Seo JH Choi CW Kim BS Shin SW Kim YH Kim JS Serum vascular endothelial growth factor per platelet count in hepatocellular carcinoma: correlations with clinical parameters and survival Jpn J Clin Oncol 2004 34 184 190 10.1093/jjco/hyh039 15121753

[B43] Leibovitch I Ben Chaim J Raviv G Mor Y Avigad I Goldwasser B Quantitative changes in platelet counts following minor urological pelvic surgery Eur Urol 1993 24 350 354 8262101 10.1159/000474327

[B44] Pedersen LM Milman N Prognostic significance of thrombocytosis in patients with primary lung cancer Eur Respir 1996 9 1826 1830 10.1183/09031936.96.09091826 8880098

[B45] Holme PA Solum NO Brosstad F Roger M Abdelnoor M Demonstration of platelet derived microvesicles in blood from patients with activated coagulation and fibrinolysis using a filtration technique and western blotting Thromb Haemost 1994 72 666 671 7900071

